# Supplementary material for: Transcriptome Analysis of Retinal and Choroidal Pathologies in Aged BALB/c Mice Following Systemic Neonatal Murine Cytomegalovirus Infection
Source: Int J Mol Sci. 2023 Feb 21;24(5):4322. doi: 10.3390/ijms24054322 (PMC10001583; doi:10.3390/ijms24054322)
Supplement: Supplementary file 1 [file ijms-24-04322-s001.zip › ijms-2210224-supplementary.pdf]

Table S1. Differentially expressed genes (DEGs) in latently infected eyes, compared to uninfected control eyes.

GeneName	Fold Change	q value
Ccdc24	-6.11	5.79E-04
Pdzph1	-5.67	4.31E-04
Apob	-5.22	1.42E-05
Sntg2	-5.13	1.67E-05
Glb1l3	-4.64	9.78E-05
Alb	-4.57	9.31E-09
Nr2e3	-4.15	5.02E-06
Nrl	-4.04	1.94E-03
Pdc	-4.01	8.06E-06
Cabp4	-3.87	1.23E-06
Wdr31	-3.85	2.71E-04
Pde6b	-3.84	6.23E-04
Gnat1	-3.83	6.81E-04
Kcnv2	-3.83	1.34E-04
Kcnj14	-3.77	8.52E-04
Pde6g	-3.63	4.37E-04
Guca1a	-3.57	1.40E-03
A930003A15Rik	-3.49	7.64E-05
Tnfaip3	-3.48	4.29E-08
Prph2	-3.45	2.20E-03
6530402F18Rik	-3.44	2.61E-06
Pde6a	-3.43	1.37E-03
Lbhd1	-3.42	1.86E-02
Rho	-3.4	5.64E-03
Rdh12	-3.38	1.07E-02
Gucy2f	-3.35	6.12E-05
AI847159	-3.33	4.37E-04
Cngb1	-3.33	4.98E-03
Cnga1	-3.32	1.02E-04
Smug1	-3.31	8.50E-04
Wdr78	-3.31	9.14E-04
Ppef2	-3.27	3.86E-08
Rom1	-3.26	1.52E-03
Bbs5	-3.25	4.58E-08
Fabp12	-3.21	6.16E-03
Nxnl1	-3.21	1.30E-02
Ano2	-3.2	7.64E-07
Gm5512	-3.19	9.58E-03
Rp1	-3.19	4.33E-04
Rgs9bp	-3.18	2.63E-04
Cpa3	-3.17	1.60E-03
Guca1b	-3.16	2.21E-02
Gngt1	-3.15	1.20E-03
Vax2os	-3.14	1.48E-04

Rp1l1	-3.13	8.33E-03
Grtp1	-3.12	5.68E-03
Nipal1	-3.08	3.82E-02
Prcd	-3.06	4.02E-06
Cdr2	-3.05	3.58E-09
Ush2a	-3.04	9.19E-03
Serinc4	-3.04	3.88E-02
Ppp1r42	-3.01	1.52E-02
Reep6	-3.01	4.56E-03
Fscn2	-3	4.94E-04
Mpp4	-2.99	1.85E-02
Ccdc126	-2.95	4.13E-04
Pex5l	-2.93	3.62E-10
Drd4	-2.92	1.72E-02
Igsf9	-2.91	2.13E-02
Rnf207	-2.88	8.00E-04
Fam57b	-2.88	3.12E-02
Wdr17	-2.86	1.02E-04
Iqcg	-2.85	3.60E-04
Rtbdn	-2.84	4.01E-04
Susd3	-2.83	1.02E-02
Slc24a1	-2.83	0.0000362
Slc16a6	-2.81	1.02E-02
Rcvrn	-2.81	2.68E-03
Spata1	-2.81	1.58E-06
Vtn	-2.79	7.09E-03
Lca5	-2.79	1.15E-09
Crocc	-2.78	1.07E-08
Mak	-2.77	2.84E-06
Fam227a	-2.77	3.11E-03
Grk1	-2.77	9.53E-03
Osbp2	-2.74	1.09E-08
Dpf3	-2.73	2.63E-08
Chst3	-2.71	4.35E-04
Glmn	-2.71	1.46E-05
Mdm1	-2.70	2.04E-06
Gm15706	-2.69	4.38E-02
Unc119	-2.69	2.11E-03
Apela	-2.69	2.47E-02
Fam3c	-2.67	2.00E-17
Tulp1	-2.67	1.32E-04
Abca4	-2.67	1.52E-03
Traf3ip3	-2.64	9.64E-03
Tdrd7	-2.64	1.37E-08
Bbs9	-2.63	5.21E-07
Fam161a	-2.62	5.02E-06
Rdh8	-2.60	2.62E-02

Ccdc113	-2.60	9.72E-03
Kcnb1	-2.59	3.97E-08
Samd7	-2.58	1.79E-02
Pla2r1	-2.56	1.24E-02
Dgke	-2.56	1.99E-12
Rabgef1	-2.55	1.65E-08
Sirt4	-2.55	9.74E-03
Rd3	-2.55	6.61E-04
Tmem181c-ps	-2.54	1.88E-04
Arr3	-2.53	2.12E-02
Pole2	-2.53	3.80E-02
Csmd1	-2.52	1.63E-05
Rmdn3	-2.52	1.54E-11
Impg1	-2.50	2.77E-02
Ankrd33b	-2.50	1.39E-07
Col4a3	-2.49	1.53E-09
Faim	-2.49	5.44E-10
Pfkfb2	-2.49	2.18E-12
Uckl1	-2.47	7.92E-04
Elovl4	-2.47	4.09E-08
Slc4a7	-2.46	3.24E-08
Herc3	-2.45	1.90E-11
Katnal2	-2.44	2.60E-02
Elovl2	-2.44	7.91E-04
Arl4d	-2.42	1.01E-02
Gnat2	-2.41	5.31E-04
Rs1	-2.41	4.73E-02
Bbs7	-2.41	3.24E-08
Arhgap39	-2.41	3.45E-02
A930015D03Rik	-2.40	9.74E-03
Col27a1	-2.40	4.11E-02
Sag	-2.39	1.47E-02
Ccdc173	-2.39	1.96E-06
Slc25a25	-2.38	1.49E-05
Pcare	-2.38	3.95E-02
Fam149a	-2.35	6.41E-06
Cdhr1	-2.34	1.62E-02
Crx	-2.34	1.96E-06
Dusp14	-2.34	4.14E-02
Ppp3cc	-2.34	3.15E-11
E230016M11Rik	-2.32	1.26E-02
Trpc1	-2.32	1.03E-07
Icmt	-2.32	3.26E-08
Lrit2	-2.31	2.99E-06
Cdan1	-2.31	5.00E-03
Bst1	-2.30	3.58E-02
A430035B10Rik	-2.30	1.43E-02

Fsd1l	-2.29	6.86E-05
BC065397	-2.29	3.92E-02
Tube1	-2.28	1.48E-03
Cplx4	-2.27	2.09E-06
Armc9	-2.27	2.32E-04
Nadk2	-2.27	2.05E-07
Dnah7b	-2.26	2.18E-05
Wisp1	-2.26	1.22E-04
Sfxn2	-2.26	4.92E-04
Pcmtd2	-2.25	1.24E-06
Usp33	-2.25	6.48E-16
Plcd3	-2.24	1.76E-03
Ric8b	-2.24	9.62E-12
D430042O09Rik	-2.23	6.07E-05
Rpgrip1	-2.23	3.85E-02
Kpna2	-2.23	3.10E-09
Rad51c	-2.21	8.91E-03
Glb1l2	-2.20	1.52E-04
5430419D17Rik	-2.20	9.90E-03
Adgrv1	-2.19	6.48E-08
Gpsm2	-2.18	2.61E-06
Vopp1	-2.18	1.15E-09
Nt5e	-2.17	1.15E-09
Pla2g7	-2.17	1.06E-04
Ip6k2	-2.17	2.04E-03
Lpcat1	-2.16	4.49E-05
Elfn1	-2.16	1.94E-03
Sec14l2	-2.16	1.45E-05
Arl13b	-2.16	6.28E-05
Spc25	-2.15	1.22E-08
Gucy2e	-2.15	3.95E-04
Peli3	-2.14	4.71E-04
Cacna1f	-2.14	9.84E-04
C1ql3	-2.14	9.55E-05
43891	-2.13	5.15E-05
Rgs9	-2.13	3.22E-07
Rrp1b	-2.13	2.04E-03
Ptp4a3	-2.13	1.02E-04
Kdm4c	-2.12	5.96E-08
Ppp2r2b	-2.12	7.83E-04
Map10	-2.12	4.83E-03
Gnb1	-2.12	2.25E-05
Gnb5	-2.12	1.84E-05
D1Ertd622e	-2.11	1.07E-09
2810029C07Rik	-2.10	3.62E-03
Glcci1	-2.08	8.01E-11
Stx3	-2.08	6.37E-09

1110002L01Rik	-2.08	5.69E-04
Pde6c	-2.06	1.73E-02
Taf4b	-2.06	4.89E-03
Zscan29	-2.06	8.90E-03
Tma7	-2.06	3.84E-04
Llgl2	-2.05	1.43E-02
Mir124a-1hg	-2.05	7.26E-07
Tbcc	-2.04	2.48E-02
Skida1	-2.04	4.59E-03
Lyar	-2.04	4.48E-09
Klhl18	-2.04	2.24E-04
Heatr5a	-2.03	3.09E-05
Taf5	-2.02	2.77E-04
Cds1	-2.02	8.96E-03
Agpat3	-2.01	4.95E-05
Tmem136	-2.01	1.27E-02
Gm8008	-2.01	2.49E-03
Plekhf2	-2.00	2.18E-10
Rbm12b1	-2.00	1.90E-02
Epb41l2	-2.00	1.85E-11
Fhod3	-2.00	6.86E-05
Crlf3	-2.00	1.20E-05
Slc16a1	-2.00	1.31E-04
H2-D1	2.01	4.02E-04
F5	2.02	2.43E-04
Ldlrap1	2.02	3.81E-02
Cenpf	2.03	2.74E-04
Mxd1	2.03	1.62E-03
Tubb6	2.06	4.67E-02
Marcksl1	2.06	2.22E-02
C4b	2.07	6.08E-07
Slfn8	2.07	2.72E-03
Ace	2.08	7.99E-03
Mid1	2.08	4.71E-04
B2m	2.09	3.63E-03
Mafb	2.10	3.75E-02
Eppk1	2.10	7.98E-03
Id1	2.11	8.97E-04
Sulf2	2.12	1.83E-02
Aspm	2.13	1.06E-03
B3galt5	2.14	2.35E-02
Tgfa	2.14	4.94E-04
Vcan	2.14	1.02E-04
Gpc4	2.14	3.90E-02
Pdlim3	2.15	1.26E-03
Serpina3n	2.17	2.48E-05
Lyn	2.17	2.71E-02

Greb1	2.18	1.35E-02
Mpeg1	2.18	1.57E-02
Mis18bp1	2.18	1.15E-02
Tcap	2.21	8.56E-03
Tnfsf10	2.21	7.90E-05
Mmp14	2.22	2.58E-05
Sp140	2.23	2.76E-02
Cd68	2.25	4.07E-02
Gbp4	2.29	1.55E-03
Tpx2	2.30	1.40E-03
Dhcr7	2.30	2.43E-02
Ets2	2.31	2.15E-02
Bcl2l15	2.34	2.63E-02
Spink5	2.35	1.78E-02
Cybb	2.35	2.32E-02
Ccl11	2.39	3.45E-02
Hcar2	2.39	3.73E-02
Cep55	2.42	2.83E-02
Prelid2	2.42	2.95E-02
Knl1	2.45	8.93E-03
Ppp1r1b	2.50	3.72E-02
Zyx	2.51	4.54E-02
Nhsl1	2.52	7.14E-03
Sgo1	2.55	4.22E-02
Serpinb10	2.55	1.10E-02
Glis3	2.57	1.65E-02
Dsg2	2.62	6.13E-05
Igtp	2.62	1.01E-02
Cd84	2.63	3.53E-02
Pkib	2.63	2.29E-02
Col18a1	2.65	1.32E-04
Gbp6	2.69	4.45E-02
Slc7a11	2.70	6.01E-12
Tlr2	2.70	1.38E-03
S1pr5	2.70	9.19E-03
Areg	2.74	4.14E-03
Gbp8	2.77	1.64E-02
Trhr	2.79	1.01E-02
Myoz2	2.82	4.56E-03
Ttc12	2.82	1.45E-02
Tmem106a	2.85	3.88E-02
Tlr6	2.88	5.91E-03
Creb5	2.91	2.50E-02
Ankrd1	2.98	4.51E-03
Gbp10	2.99	6.21E-04
Ace2	3.08	2.86E-02
Myh6	3.09	3.57E-02

Tnfaip2	3.09	2.22E-02
Snrpn	3.10	5.75E-08
Lcp1	3.18	4.07E-03
Upb1	3.19	1.09E-02
Nupr1	3.21	2.16E-08
Casp12	3.27	6.39E-07
Tlr13	3.28	4.33E-02
Mctp1	3.29	2.01E-02
Bub1	3.36	1.62E-02
Adtrp	3.44	6.54E-13
Ccnb1	3.50	8.77E-04
Dchs2	3.55	5.35E-03
Pik3cg	3.72	3.29E-02
Masp1	3.78	1.64E-02
Kcnj15	3.84	1.88E-03
Arnt2	3.93	1.31E-03
Cfi	4.12	2.42E-03
C4a	4.14	1.40E-04
Zhx2	4.19	1.71E-04
Tfcp2l1	4.19	7.91E-04
Cytip	4.27	4.65E-02
Col3a1	4.42	3.16E-02
Tgtp1	4.45	2.33E-02
Pde1a	4.52	2.00E-17
F830016B08Rik	4.53	3.22E-02
Apobr	5.26	3.62E-03
Tgtp2	5.56	5.88E-04
Nlrc5	5.84	1.83E-02
Msr1	8.18	3.83E-03
Gm4841	8.50	4.24E-04
Gm4951	9.12	3.47E-07
Ccr1	11.07	1.96E-02
Sbsn	17.02	2.00E-02
Mmp13	20.47	1.09E-02
Stfa2l1	25.67	3.49E-02
S100a9	28.44	1.62E-03
Krt16	38.81	4.24E-03
Eif2s3y	381.85	2.11E-05
Ddx3y	511.83	5.77E-05
Uty	967.51	3.60E-04
Kdm5d	2214.91	1.17E-02
Gm14308	11202567.26	3.56E-09

Table S2. Differentially expressed genes (DEGs)
in infected eyes with severe retinal degeneration,
compared to infected eyes with milder retinal degeneration.

GeneName	Fold Chang	q value
Kcnj1	-312.996	1.16E-03
Pira11	-174.853	1.67E-02
Vwa5b2	-127.116	4.84E-02
Pdzk1	-40.7859	1.21E-02
Gm3435	-13.737	3.16E-02
A930004D1	-6.77396	3.84E-04
E130310I04	-4.72397	2.67E-02
Sfmbt2	-4.25748	3.16E-02
Gm15706	-4	3.90E-04
Ahrr	-2.80889	2.21E-02
Guca1b	-2.73208	7.94E-04
AI847159	-2.73208	4.25E-02
Rdh12	-2.67586	5.34E-04
Fscn2	-2.65737	2.50E-03
Lbhd1	-2.60268	3.24E-02
Nxnl1	-2.56685	4.26E-04
Cabp4	-2.56685	3.02E-03
Pde6b	-2.54912	0.00E+00
Glb1l3	-2.53151	6.90E-03
Nrl	-2.47942	2.85E-04
Rho	-2.47942	3.37E-02
Gnat1	-2.36199	1.20E-05
Pde6a	-2.34567	4.60E-05
Wdr78	-2.34567	2.05E-03
Prph2	-2.31338	7.94E-04
Vtn	-2.2974	1.54E-04
Gngt1	-2.23457	0.00E+00
Samd7	-2.23457	2.34E-03
Impg1	-2.18859	1.70E-03
Pla2r1	-2.17347	1.96E-04
Guca1a	-2.17347	1.55E-02
Rom1	-2.15846	2.20E-05
Rp1	-2.14355	1.20E-05
Nr2e3	-2.14355	2.85E-04
Pdc	-2.11404	1.30E-05
Sag	-2.11404	2.55E-04
Cnga1	-2.07053	1.20E-05
Rgs9bp	-2.07053	2.67E-04
Klf5	2.013911	3.24E-02
Krt7	2.084932	3.16E-02
Cyp2f2	2.099433	3.37E-02
Muc4	2.329467	0.00E+00
Gm9573	2.462289	3.54E-03

Gbp6	2.496661	1.96E-04
Krt19	2.657372	2.19E-04
Myoc	2.675855	2.88E-02
Mmp27	128.8903	1.32E-02
Rasal3	186.1085	1.56E-02

Table S3. Primers.

Genes	Forward Primer (5'->3')	Reverse Primer (5'->3')
IL-33	GGCTCACTGCAGGAAAGTACA	TTGGTCTTCTGTTGGGATCTTCT
STAT3	GGAAAAGGACATCAGTGGCAAG	CAGGTACGGGGCAGCACTA
STAT6	GCTACTGGTCAGATCGGCTG	CAGTGAGCGAATGGACAGGT
RHO	CCCATCAACTTCCTCACGCT	AGGGCGATTTCACCTCCAAG
CREB1	AGCTTGTACCACCGGTATCC	CCATGGACCTGGACTGTCTG
GRK1	CAGCTCGAAGCCGGGATG	GCCACACATTTAGGTCCCCA
PDE6A	CATGTACAGGACCCGCAACG	GTCACAGAAATGCTCATCCTCTTC
B2m	GTATACTCACGCCACCCACC	TCTCGATCCCAGTAGACGGT
β-actin	CGTCACACTTCATGATGGAATTGA	CGGTTCCGATGCCCTGAGGCTCTT
